# Ghrelin is essential for lowering blood pressure during torpor

**DOI:** 10.3389/fendo.2024.1487028

**Published:** 2024-10-10

**Authors:** Kazuma Matsui, Takanori Ida, Kanae Oishi, Masayasu Kojima, Takahiro Sato

**Affiliations:** ^1^ Division of Molecular Genetics, Institute of Life Science, Kurume University, Fukuoka, Japan; ^2^ Division for Identification and Analysis of Bioactive Peptides, Department of Bioactive Peptides, Frontier Science Research Center, University of Miyazaki, Miyazaki, Japan

**Keywords:** blood pressure, ghrelin, torpor, circadian rhythm, body temperature

## Abstract

**Introduction:**

Daily torpor is an active hypothermic phenomenon that is observed in some mammals and birds during fasting. A decrease in blood pressure has also been observed in torpor; however, there remains a lack of knowledge of the underlying mechanism. We have previously reported that ghrelin, an orexigenic hormone, has a hypothermic effect and is essential for the induction and maintenance of torpor. It is also known that the ghrelin secretion is enhanced during fasting and that ghrelin receptors are distributed in the cardiovascular system. Therefore, this study was conducted to test the hypothesis that ghrelin is actively involved in the regulation of blood pressure during torpor induction.

**Methods:**

Male wild-type and ghrelin gene-deficient mice were generated by homologous recombination as previously reported. Mice, 10 weeks old, were included in this study and housed five per cage. The mice were maintained on a 12-h light/dark cycle (lights on from 7:00 to 19:00) with access to food and water ad libitum.

**Results:**

The continuous measurement of blood pressure using a telemetry system showed that induction of torpor by fasting did not decrease blood pressure in ghrelin gene-deficient mice. The analysis of heart rate variability revealed that sympathetic nerve activity was predominant in ghrelin-deficient mice during fasting. Furthermore, these features were cancelled by administration of a ghrelin receptor agonist and were comparable to those in wild-type mice.

**Discussion:**

In this study, we showed that blood pressure was elevated in *ghrl^-/-^* mice and that the blood pressure rhythm was abnormal. Furthermore, we showed that the ghrelin gene deficiency does not cause sufficient blood pressure reduction upon entry into the torpor, and that the administration of the ghrelin receptor agonist, GHRP-6, causes blood pressure reduction associated with torpor. Thus, we have shown for the first time that the active role of ghrelin is essential for active blood pressure reduction associated with torpor, and that this action is mediated by the inhibition of sympathetic nerve activity by ghrelin.

## 1 Introduction

Torpor is a physiological phenomenon in which an animal actively lowers its core body temperature and suppresses energy waste during food shortages. Torpor has been observed in some mammals, such as mice, and birds, such as hummingbirds, and has long been known as a survival strategy during starvation. However, the mechanism through which torpor is induced and maintained remains unclear.

Recently, we reported that ghrelin is an essential hormone for the induction and maintenance of torpor in mice (1). Ghrelin is a hormone isolated and purified from the stomach as an endogenous ligand for the GH secretagogue receptor (GHS-R) (2) and induces a positive energy balance by promoting GH secretion as well as food intake and fat accumulation through a GH-independent mechanism (3). For this reason, ghrelin secretion is enhanced in negative energy states, such as during fasting (4–8). When adult mice are subjected to fasting, the body temperature gradually decreases, and torpor is induced (9, 10). Thus, plasma concentrations of ghrelin are inversely correlated with changes in body temperature. We found that ghrelin secreted from the stomach during fasting acts on the arcuate nucleus of the hypothalamus and inhibits sympathetic activity that eventually enters the brown adipose tissue (1). As a result, the expression of UCP-1, the essence of heat production, is suppressed in brown adipose tissue, and the heat production is reduced, resulting in a decrease in body temperature (1). At the same time, ghrelin retrogradely signals through the gastric vagus nerve to the solitary nucleus to maintain a constant body temperature rhythm and torpor state (1). Thus, the activation of ghrelin signaling during fasting induces torpor and maintains a low body temperature.

In the torpor state, there is a decrease in body temperature and blood pressure (11, 12). As an excessive decrease in blood pressure can be life-threatening, it is presumed that the decrease in blood pressure observed in the torpor state is appropriately regulated by an active mechanism. However, to the best of our knowledge, little is known regarding the regulation of torpor and blood pressure. In contrast, blood pressure regulation by ghrelin has been studied, and the high expression of ghrelin receptors, GHS-R, in the heart, kidneys, and blood vessels is considered evidence that ghrelin is involved in blood pressure regulation (13–19). The circulating ghrelin concentrations have been reported to be inversely correlated with blood pressure, and acute and chronic hypotensive effects of ghrelin have been reported in normotensive animals, healthy individuals, animals and patients with heart failure, and hypertensive animals (20, 21). The mechanisms by which ghrelin regulates blood pressure appear to be related to autonomic nervous system regulation, direct vasodilation, and diuretic action of kidneys (21–24).

These facts remind us of the possibility that blood pressure regulation by ghrelin may actively contribute to the induction and maintenance of torpor. In this study, we aimed to clarify whether ghrelin is responsible for blood pressure regulation in the torpor state by comparatively analyzing the blood pressure variability in ghrelin-deficient mice.

## 2 Materials and methods

### 2.1 Experimental animals

Male wild-type (WT) and ghrelin gene-deficient (*ghrl^-/-^*) mice were generated by homologous recombination as previously reported (1). Mice, 10 weeks old, were included in this study and housed five per cage. The mice were maintained on a 12-h light-dark cycle (lights on from 7:00 to 19:00) with access to food and water ad libitum. The room temperature was maintained at 23 ± 1°C. All animal studies were handled in accordance with the Guide for the Care and Use of Laboratory Animals, as adopted by the U.S. National Institutes of Health, and the specific protocols were approved by the Institutional Animal Care and Use Committee of Kurume University School of Medicine (approval no. 2010-026, 2011-062, 2020-005, 2021-006-1, 2022-125, 2023-097, 2024-022-1).

### 2.2 Measurement of body weight, tissue wet weight, and blood pressure

Body weight, tissue wet weight, and blood pressure were measured in 12-week-old intact WT and *ghrl^-/-^* mice. Blood pressure was measured from 13:00 to 15:00 using a tail-cuff plethysmograph with a blood pressure-measuring system (MODEL MK-2000ST; Muromachi Kikai, Tokyo, Japan). The blood pressure was measured following 15 min warming at 37°C in an animal holder made of dark brown acryl, allowing blood pressure measurement under relatively stress-free conditions. Five measurements were taken for each animal and the data represented the average of three values, excluding the maximum and minimum values.

### 2.3 Hematoxylin and eosin staining

The hearts and kidneys from 12-week-old mice were quickly immersed in cold 4% paraformaldehyde/PBS. After dehydration and permeabilization, the tissue was paraffin-embedded and incised at a thickness of 5 μm. The sections were stained with a hematoxylin and eosin staining kit (Scy Tek Laboratories, UT, USA) and viewed under an optical microscope (Evident, Tokyo, Japan).

### 2.4 Measurement of aldosterone levels

Intact 12-week-old WT and *ghrl^-/-^* mice were used. Whole blood was collected by decapitation at 14:00, and serum was obtained after centrifugation. The serum aldosterone levels were measured using an ELISA kit (Cayman, Chemical Co., Ann Arbor, MI).

### 2.5 Implantation of the telemetric transmitter

The mice were anesthetized by intraperitoneal injection of a mixture of ketamine and xylazine (5 mg/kg; Dainippon Pharmaceutical, Osaka, Japan). At the age of 10 weeks, mice were separated into individual cages and implanted with the telemetry device (PA-C10; Data Sciences International, St. Paul, MN, USA) to the abdominal aorta, according to the manufacturer’s outlines. Briefly, after an incision of abdominal skin and musculature, the abdominal aorta was exposed, and the catheter of the transmitter was introduced upstream into the vessel. The transmitter body was placed subcutaneously in the left flank. After implantation, the mice were rapidly recovered with atipamezole. A minimum of 1 week was allowed for recovery and for the mice to regain their pre-surgical weight. The diurnal rhythm of blood pressure and heart rate in mice was measured 4 weeks after surgery. During the experiment, a PhysiolTel-Receiver (model RPC-1; Data Sciences International) was placed under each animal’s cage to record the blood pressure. For the diurnal rhythm of blood pressure, data were obtained every 5 min and graphed.

### 2.6 Ghrelin administration to mice

The rat ghrelin was provided by Dr. Hiroshi Hosoda (National Cerebral and Cardiovascular Center). To investigate the changes in blood pressure after ghrelin administration, the 12-week-old mice with implanted telemetric devices were administered 100 μg/kg ghrelin intraperitoneally at 7:00 or 19:00.

### 2.7 Implantation of mini-osmotic pumps

To determine the role of ghrelin in blood pressure and heart rate regulation during torpor induction, the mice implanted with telemetric devices had their blood pressure and heart rate measured while receiving GHRP-6 (Sigma, St. Louis, MO, USA), a ghrelin receptor agonist. Mini-osmotic pumps filled with GHRP-6 (Model 2002; Durect Co., Cupertino, CA, USA) were implanted subcutaneously 10 days before reaching 14 weeks of age. The mini-osmotic pumps delivered GHRP-6 at 42 ng/0.5 μl/h/g body weight for 14 days.

### 2.8 Fasting experiment

WT and *ghrl^-/-^* mice, and *ghrl^-/-^* mice receiving continuous administration of GHRP-6 with osmotic mini-pumps were all initiated on a 48-h fast at the end of the light phase (19:00) when they reached 14 weeks of age. During this period, they were free to drink, and after 48 h, they were fed again. To better understand the changes in blood pressure and heart rate during fasting, we calculated the difference in mean blood pressure (ΔBP) and mean heart rate (ΔHR). First, the average of 12 data obtained during the first hour of fasting (18:00-18:55) was determined and set as the reference value, or zero. Next, the data acquired during all fasting and refeeding periods were used to calculate the mean value of the 12 data obtained every hour. These values were subtracted from the reference value to obtain the difference (ΔBP and ΔHR, respectively).

### 2.9 Heart rate variability analysis

The heart rate variability (HRV) was measured to assess the autonomic function (25). The data were recorded by electrocardiography using a PowerLab system and analyzed using the LabChart software (version 6.0; AD Instruments Pty Ltd., Australia). The frequency-domain variables included: low (LF, 0.04–0.15 Hz) and high (HF, 0.15–0.4 Hz) frequencies. Subsequently, the LF/HF ratio was calculated.

### 2.10 Statistical analysis

The data are displayed as mean ± S.E.M. The significance of the differences within each group was determined using one-way ANOVA, followed by Bonferroni’s *post-hoc* test. When two groups were compared, the Student’s *t*-test or two-way ANOVA followed by Bonferroni’s *post-hoc* test was used. The analyses were performed using the Prism software (version 5.0; GraphPad Software, San Diego, CA, USA); p < 0.05 was considered to be significant.

## 3 Results

### 3.1 *Ghrl^-/-^* mice have higher blood pressure

**Figure 1:**
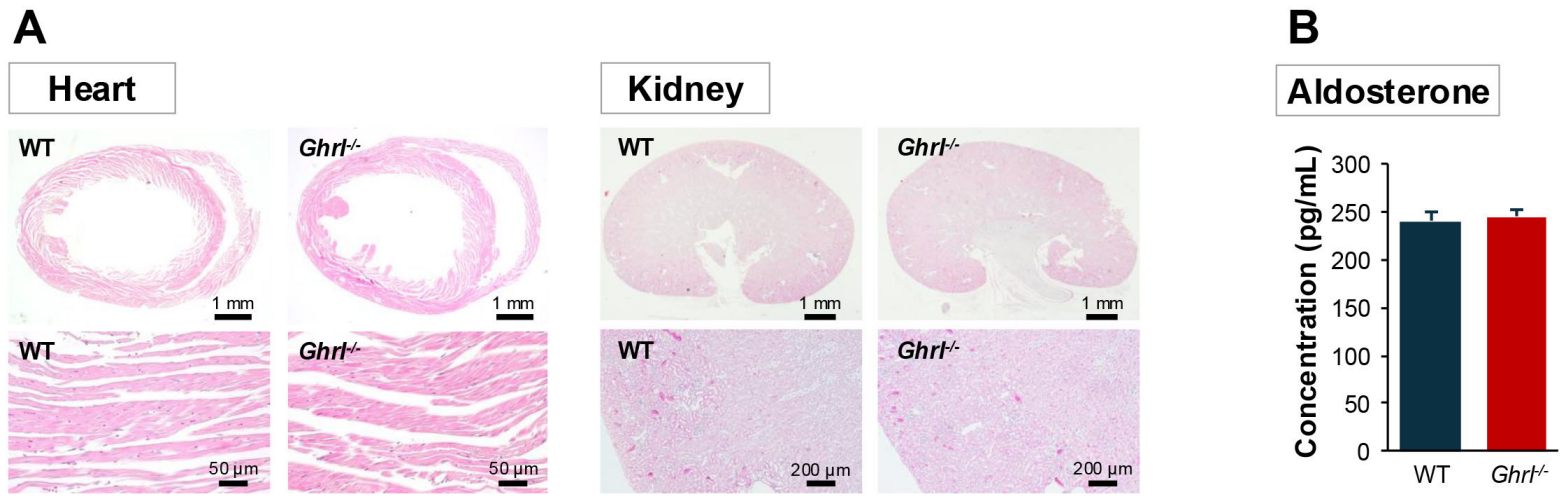
Histology and hormonal secretion of the circulatory regulatory system in *ghrl^-/-^* mice. **(A)** Hematoxylin-eosin stained images of heart and kidney. **(B)** Basal secretion of serum aldosterone in *ghrl^-/-^* mice.

To determine the effect of ghrelin deficiency on blood pressure, we measured the blood pressure in *ghrl^-/-^* mice using tail-cuff plethysmography and found that blood pressure was higher in *ghrl^-/-^* mice than in WT mice (**Table 1**). Consistent with previous reports (26, 27), ghrelin deficiency did not cause any changes in body weight, and there were no differences in heart and kidney tissue wet weight, body weight ratio, or histology (**Table 1**, **Figure 1A**). Since blood pressure is known to be regulated by humoral and neural factors, we next investigated whether ghrelin deficiency affects the basal state of humoral factors. The renin-angiotensin-aldosterone system, vasopressin, and atrial natriuretic peptide are known as humoral factors that regulate blood pressure, and the renin-angiotensin-aldosterone system has been implicated in blood pressure regulation by ghrelin (28). Therefore, we measured serum aldosterone levels and found no differences between the two groups (**Figure 1B**), suggesting that the kidney function was normal despite the ghrelin gene deficiency. In other words, the humoral factors that regulate blood pressure were shown to be normal despite ghrelin deficiency.

**Table 1:** Body weight, organ weights, and blood pressure in *ghrl^-/-^* mice.

	WT mice	*Ghrl^-/-^* mice
Body weight (g)	31.8 ± 1.8	30.8 ± 2.3
Blood pressure (mmHg)	106.8 ± 9.6	135.8 ± 7.1 ^*^
Heart weight (mg)	165.6 ± 19.9	162.8 ± 12.5
Relative heart weight (/100 g body weight)	519.5 ± 38.2	529.7 ± 40.6
Kidney weight (mg)	256.4 ± 11.3	251.8 ± 22.2
Kidney relative weight (/100 g body weight)	807.1 ± 25.9	817.2 ± 24.1

Data are presented as means ± SEM (n = 5); ^*^P < 0.05 versus WT mice.

### 3.2 Ghrelin-gene deficiency significantly increased the blood pressure in the light phase

**Figure 2:**
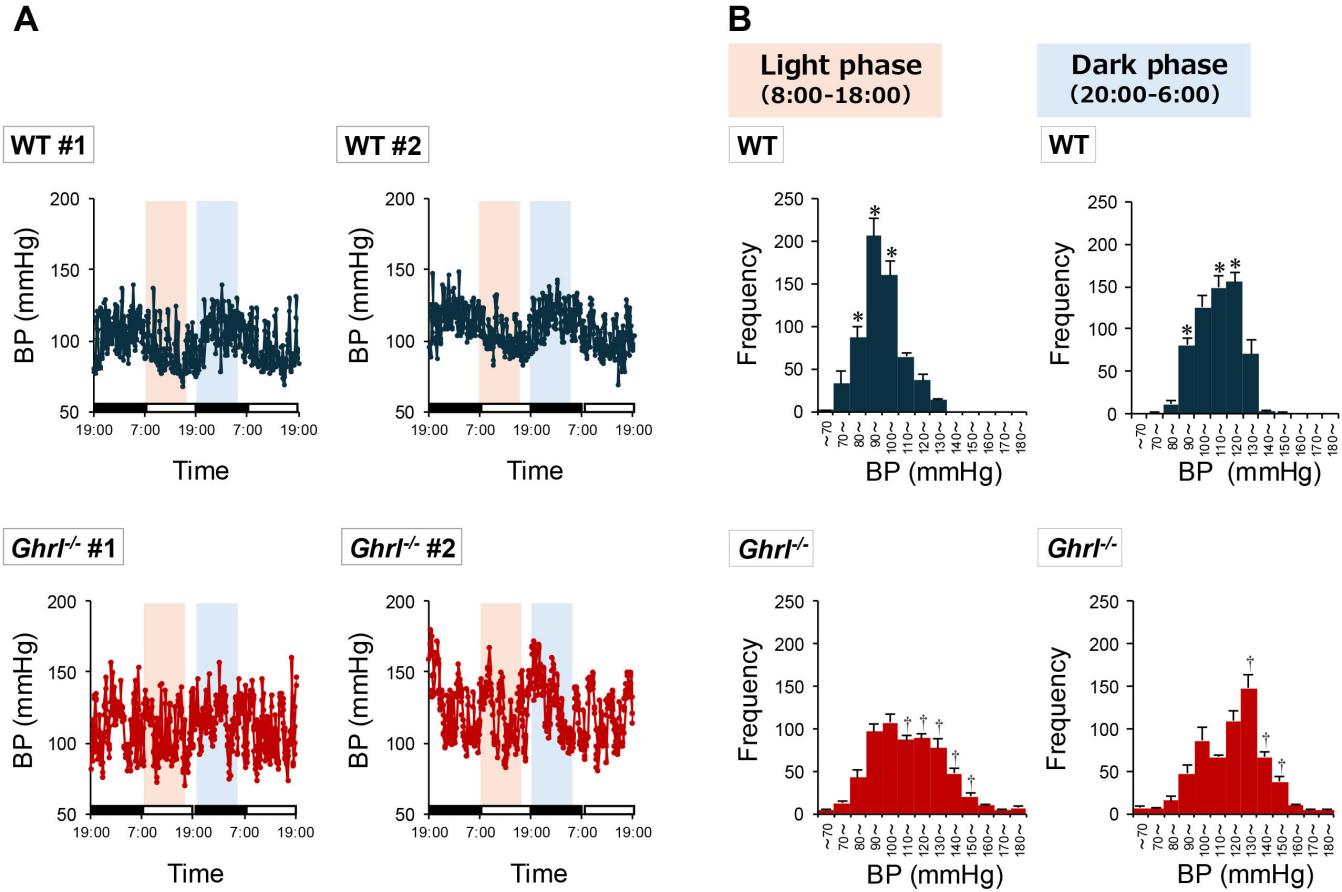
Diurnal rhythm of blood pressure in *ghrl^-/-^* mice. **(A)** Two-day blood pressure changes for representative individual mice. The black bar shown on the horizontal axis represents the dark phase, and similarly the white bar represents the light phase. The orange and light blue backgrounds shown in the graph indicate the range over which data were used to draw the histogram in **(B)**. **(B)** Histograms of blood pressure during the light phase (8:00 - 18:00; data obtained from orange background in **(A)**) and dark phase (20:00 - 6:00 the next day; data obtained from light blue background in **(A)**) of *ghrl^-/-^* mice. Data are means ± SEM (n = 5). ^*^P<0.05 (higher frequency in WT mice compared to *ghrl^-/-^* mice), ^†^P<0.05 (higher frequency in *ghrl^-/-^* mice compared to WT mice).

To determine whether blood pressure in *ghrl^-/-^* mice was always high, the blood pressure was continuously measured using an automated telemetry system. A clear diurnal rhythm was observed in the blood pressure of WT mice, which was higher during the dark phase, the active phase of nocturnal mice, and lower during the light phase, the resting phase of nocturnal mice (**Figure 2A**). In contrast, the blood pressure of *ghrl^-/-^* mice was indistinct and was generally higher than that of WT mice (**Figure 2A**). Such diurnal variation in blood pressure was also consistent with diurnal variation in heart rate (**Supplementary Figure 1**). To quantitatively evaluate the blood pressure data of WT and *ghrl^-/-^* mice, histograms were generated from the data of light (8:00-18:00) and dark (20:00-6:00) phases (**Figure 2B**), and mean and median values were calculated (**Table 2**). The blood pressure in both WT and *ghrl^-/-^* mice was higher during the dark phase of activity than during the light phase of rest, indicating that the central clock was normal. Both mean and median blood pressure were higher in *ghrl^-/-^* mice only during the light phase (**Table 2**). Thus, the ghrelin gene deficiency prevented blood pressure reduction during the light phase, which is the resting phase in nocturnal mice.

**Table 2:** Mean and median blood pressure in *ghrl^-/-^* mice.

	WT mice	*Ghrl^-/-^* mice
Mean blood pressure		
Light phase	99.8 ± 3.1	116.4 ± 4.2^*^
Dark phase	115.2 ± 2.8	124.7 ± 4.1
Median blood pressure		
Light phase	97.2 ± 3.0	113.9 ± 5.3^*^
Dark phase	117.5 ± 3.4	124.9 ± 5.3

Each was calculated from the data used to create the histograms in **Figure 2B**. Data are presented as means ± SEM (n = 5); ^*^P < 0.05 versus WT mice.

### 3.3 Ghrelin induces a decrease in blood pressure

**Figure 3:**
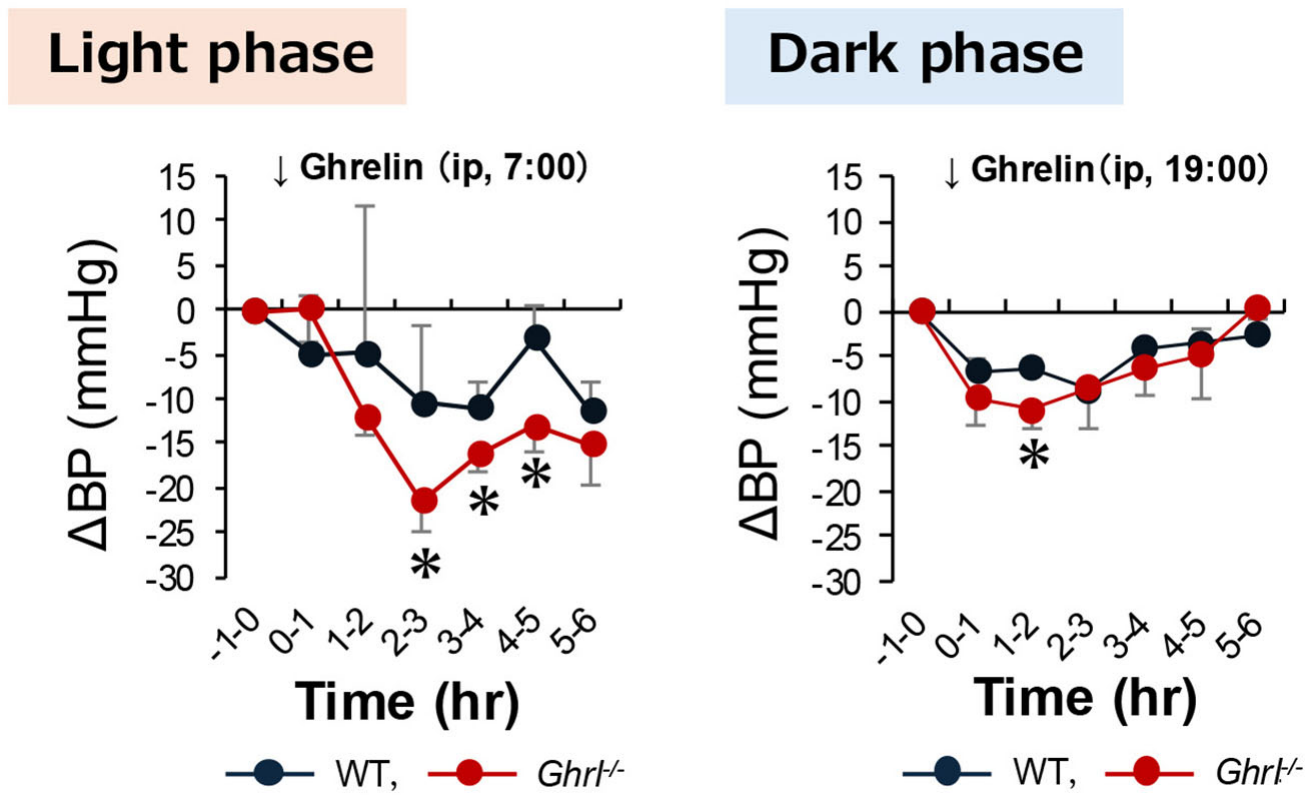
Blood pressure changes in *ghrl^-/-^* mice following peripheral ghrelin administration. Ghrelin was administered intraperitoneally to WT mice and *ghrl^-/-^* mice at 7:00, the start of the light phase, and at 19:00, the start of the dark phase. The graphs show the range of change in blood pressure compared to that before ghrelin administration. Data are means ± SEM (n = 5). ^*^P<0.05 versus WT mice.

Subsequently, we analyzed the effects of ghrelin on blood pressure reduction in WT and *ghrl^-/-^* mice during the light and dark phases. Ghrelin induced a transient decrease in blood pressure in all the groups (**Figure 3**). The ghrelin-induced reduction in blood pressure was greater in *ghrl^-/-^* mice than in WT mice (**Figure 3**). In particular, the blood pressure was markedly reduced in *ghrl^-/-^* mice treated with ghrelin during the light phase (**Figure 3**). These results suggest that even *ghrl^-/-^* mice with high blood pressure can have a lower blood pressure.

### 3.4 The ghrelin-ghrelin receptor system is required for active blood pressure reduction during torpor

**Figure 4:**
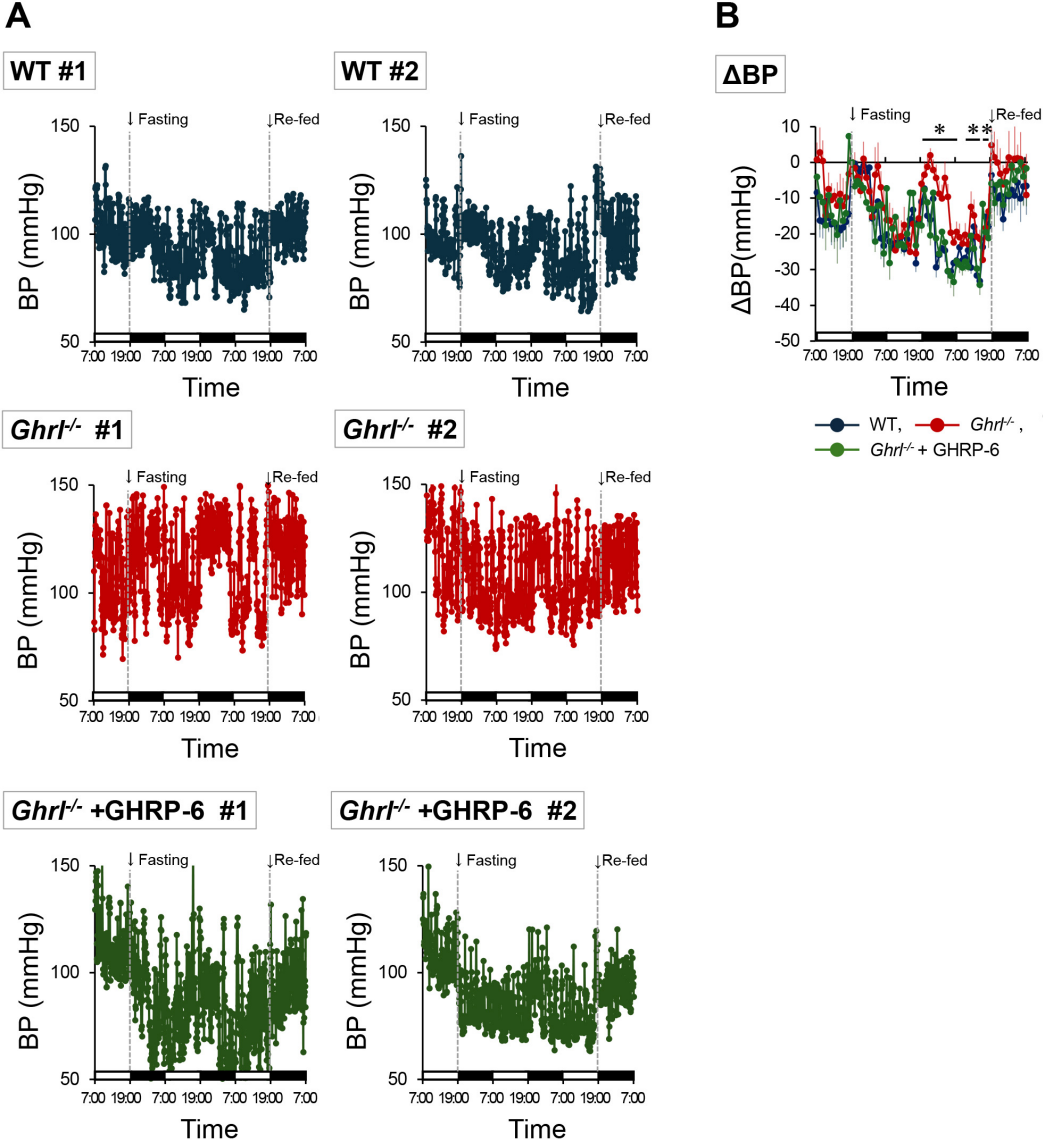
Blood pressure changes in *ghrl^-/-^* mice following fasting. Ghrl^-/-^+GHRP-6 indicates that the *ghrl^-/-^* mice were administered the ghrelin receptor agonist, GHRP-6, with an osmotic mini-pump, and Re-fed indicates re-feeding. The black bar shown on the horizontal axis represents the dark phase, and similarly the white bar represents the light phase. **(A)** Blood pressure changes after fasting in representative individual mice. **(B)** Range of change in hourly means blood pressure data from the start of fasting. Data are means ± SEM (n = 5). *P<0.05 (comparison between WT mice, *ghrl^-/-^* mice, and *ghrl^-/-^* mice treated with the ghrelin receptor agonist GHRP-6).

Ghrelin secretion in mice is maximal during fasting, when torpor is induced (1). Therefore, we measured the blood pressure and heart rate in *ghrl^-/-^* mice during torpor induction. In WT mice, a decrease in blood pressure was observed with the passage of fasting time while maintaining the light-dark cycle, and the decrease in blood pressure was cancelled by re-feeding (**Figure 4A**). In *ghrl^-/-^* mice, the individual differences in blood pressure fluctuation patterns were large, and large blood pressure fluctuations were observed in short cycles. However, the overall trend was that the blood pressure remained high during the fasting period (**Figure 4A**). To determine whether the blood pressure abnormalities observed in *ghrl^-/-^* mice could be improved by activation of the ghrelin receptor system, we performed fasting experiment in *ghrl^-/-^* mice implanted with an osmotic mini-pump filled with the ghrelin receptor agonist GHRP-6 and found that the blood pressure in *ghrl^-/-^* mice was improved to the level of WT mice (**Figure 4A**). To quantitatively evaluate these observations, we calculated the mean blood pressure 24-48 hours after the onset of fasting and found that it was higher only in *ghrl^-/-^* mice, with no difference between WT mice and *ghrl^-/-^* mice pretreated with GHRP-6: WT mice: 98.7 ± 3.7 mmHg, *ghrl^-/-^* mice: 108.2 ± 2.2 mmHg, *ghrl^-/-^* +GHRP-6 mice: 89.9 ± 4.8 mmHg. In addition, we calculated hourly average data and shown as blood pressure changes from the start of fasting (**Figure 4B**). WT mice and *ghrl^-/-^* mice pretreated with GHRP-6 showed similar blood pressure trends, with a low value at approximately 36 h after the start of fasting during torpor induction (**Figure 4B**). In contrast, fasting did not produce a sufficient decrease in blood pressure in *ghrl^-/-^* mice (**Figure 4B**). In all groups, the blood pressure increased quickly after re-feeding; however, this increase was greater in *ghrl^-/-^* mice (**Figure 4B**). These results showed that the decrease in blood pressure seen during torpor does not occur in *ghrl^-/-^* mice. In addition, it was shown that preadministration of GHRP-6 using an osmotic mini-pump can reduce the mean blood pressure of *ghrl^-/-^* mice to the same level as that of WT mice. The change in heart rate during fasting was also similar to the change in blood pressure, but differences between the *ghrl^-/-^* mice group and the other two groups were observed from an earlier time from the start of fasting (**Supplementary Figure 2**). These results suggest that activation of the ghrelin-ghrelin receptor system is required for active blood pressure reduction during torpor induction.

### 3.5 Inhibition of sympathetic nerve activity by the ghrelin-ghrelin receptor system is necessary for blood pressure reduction during fasting

**Figure 5:**
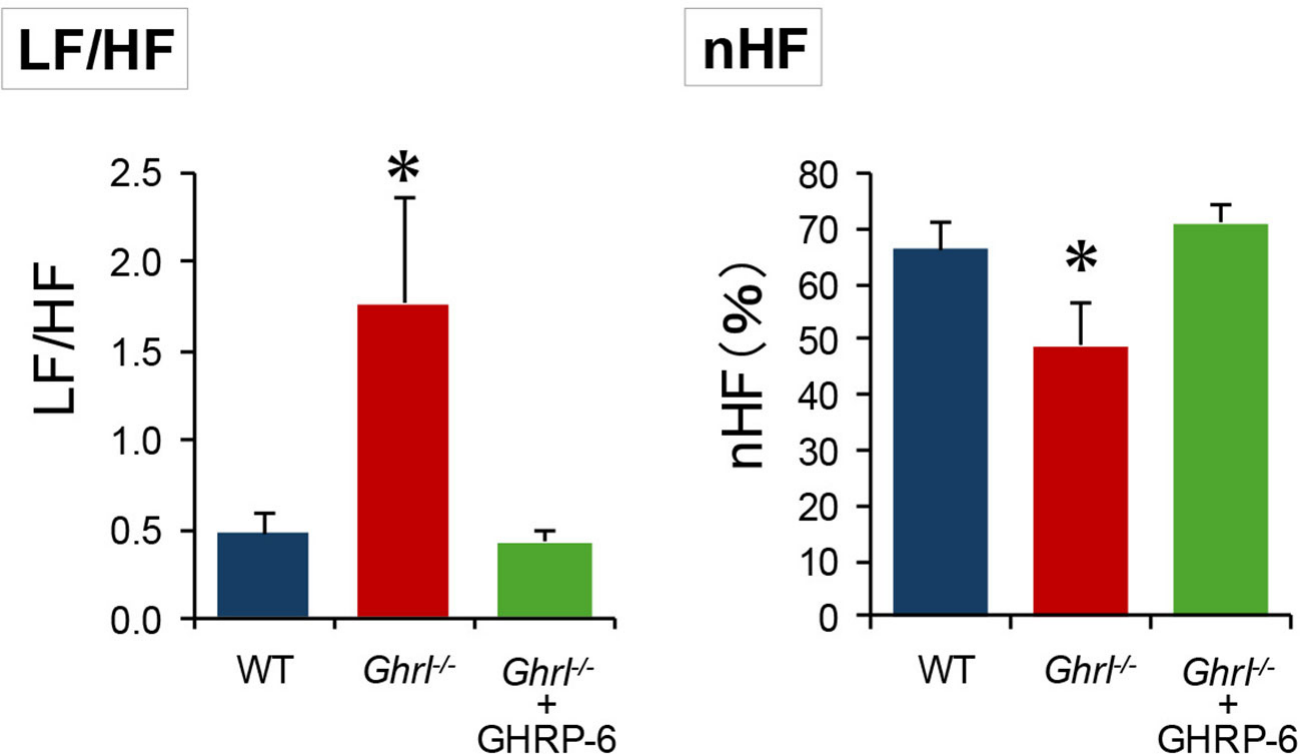
Analysis of heart rate variability in *ghrl^-/-^* mice during fasting. Heart rate variability analysis 36 h after the start of fasting. The left panel shows LF/HF ratio, a measure of sympathetic activity, and the right panel shows nHF, a measure of parasympathetic activity. *Ghrl^-/-^* +GHRP-6 indicates that *ghrl^-/-^* mice were administered the ghrelin receptor agonist, GHRP-6, with an osmotic mini-pump. ^*^P<0.05 (comparison between WT mice, *ghrl^-/-^* mice, and *ghrl^-/-^* mice treated with the ghrelin receptor agonist GHRP-6).

As there were no morphological or endocrinological abnormalities in the heart or kidneys of *ghrl^-/-^* mice (**Figure 1**), we analyzed the autonomic nervous system activity to understand the mechanism by which ghrelin lowered blood pressure during torpor induction. Analysis of HRV is widely used as a standard method for assessing autonomic nervous functions (25). Particularly, LF and HF spectral components of HRV are used as the separate metrics of sympathetic and parasympathetic functions (25). The LF/HF ratio, a measure of sympathetic activity, was higher and normalized HF% (nHF%), a measure of parasympathetic activity, was lower in *ghrl^-/-^* mice after 36 h of fasting (**Figure 5**). Furthermore, these changes were cancelled in *ghrl^-/-^* mice pretreated with GHRP-6 using an osmotic mini-pump and were comparable to those in WT mice (**Figure 5**). Taken together, these results indicated that blood pressure is higher in *ghrl^-/-^* mice because of significant sympathetic activity due to ghrelin deficiency and that this effect is suppressed by the activation of the ghrelin-ghrelin receptor system. Thus, it was suggested that the suppression of the sympathetic nerve activity by activation of the ghrelin-ghrelin receptor system is necessary during torpor induction.

## 4 Discussion

In this study, we showed that the blood pressure was elevated in *ghrl^-/-^* mice and that the blood pressure rhythm was abnormal. Furthermore, we showed that the ghrelin gene deficiency does not cause sufficient blood pressure reduction upon entry into the torpor, and that the administration of the ghrelin receptor agonist, GHRP-6, causes blood pressure reduction associated with torpor. Thus, we have shown for the first time that the active role of ghrelin is essential for active blood pressure reduction associated with torpor, and that this action is mediated by the inhibition of sympathetic nerve activity by ghrelin.

The effects of ghrelin on blood pressure regulation have been thoroughly investigated, with many reports from different perspectives (16, 18, 20–24). For example, the intravenous injection of ghrelin in healthy subjects produces a relatively long-lasting decrease in blood pressure without any change in heart rate (14, 18). This ghrelin-induced decrease in blood pressure is thought to be mediated by the action of ghrelin on the solitary nucleus, rather than by its direct action on the cardiovascular system (29). This report confirms that ghrelin infusion into the solitary nucleus causes a decrease in blood pressure and heart rate as well as an inhibitory effect on the sympathetic nervous system (29). Thus, the common consensus is that ghrelin exerts its blood pressure-lowering effects; however, all these reports are based on pharmacological studies. Therefore, the analysis of the role of ghrelin in the physiological phenomenon of torpor, which is accompanied by a decrease in active body temperature and blood pressure, has been delayed.

In this study, we continuously measured blood pressure in *ghrl^-/-^* mice and showed for the first time that blood pressure does not decrease in *ghrl^-/-^* mice, even during the light period and fasting, when blood pressure should decrease, and that the amplitude of the blood pressure rhythm is large. We also showed that these abnormalities were reversed by the administration of GHRP-6, a ghrelin receptor agonist. In other words, we showed that ghrelin has a blood pressure-lowering effect even at the physiological level and that it is necessary for maintaining blood pressure rhythm.

The circadian rhythms triggered by the central biological clock strictly regulate various physiological functions of the body, including blood pressure (30, 31). We previously examined free-running cycles from actograms generated by an infrared behavior-measuring device and found no differences between WT and *ghrl^-/-^* mice (1). We also analyzed the expression cycles of key clock genes, such as *Per2* (period circadian 2) and *Clock* (clock circadian regulator), in the suprachiasmatic nucleus of the hypothalamus and found no differences between WT and *ghrl^-/-^* mice (1). These facts indicate that the loss of the ghrelin gene does not cause abnormalities in the central biological clock.

However, ghrelin deficiency causes abnormal circadian rhythms of body temperature and prevents torpor during fasting (1). In this study, blood pressure fluctuations in *ghrl^-/-^* mice were also large, indicating that the circadian rhythm of blood pressure was unstable while maintaining a light/dark cycle. Although this study did not investigate the detailed mechanism related to blood pressure rhythm abnormalities associated with ghrelin gene deficiency, we were able to clearly demonstrate that ghrelin deficiency causes high blood pressure and abnormal blood pressure rhythms. Furthermore, when the effect of the ghrelin receptor agonist GHRP-6 on blood pressure is considered, it is suggested that ghrelin plays an active role in the reduction of blood pressure that occurs during torpor induction.

We therefore conducted an experiment in which ghrelin was administered to WT and *ghrl^-/-^* mice. We observed a decrease in blood pressure in all groups; however, the magnitude of blood pressure reduction differed between the groups, with a greater decrease in *ghrl^-/-^* mice than in WT mice. These results confirmed the previous findings that exogenous ghrelin administration lowers blood pressure (18) and indicated that *ghrl^-/-^* mice can also lower blood pressure with ghrelin supplementation alone. In addition, a greater decrease in blood pressure was observed in *ghrl^-/-^* mice during the light phase than during the dark phase. As previously reported, plasma ghrelin secretion was higher during the light period and lower during the dark period (1). In other words, ghrelin secretion and blood pressure are inversely correlated, and ghrelin deficiency increases blood pressure during the light period. This may be because exogenous ghrelin administration acts more effectively during the light phase than during the dark phase. These results indicate that the blood pressure-lowering mechanism downstream of the ghrelin receptor is normal despite the absence of the ghrelin gene, and that *ghrl^-/-^* mice retain the ability to lower blood pressure with ghrelin.

To clarify one aspect of the mechanism by which ghrelin actively lowers blood pressure during fasting, the HRV analysis was performed 36 h after fasting, when the body temperature is known to decrease the most during torpor induction (1). *Ghrl^-/-^* mice have a high LF/HF ratio, a measure of sympathetic activity, and are sympathetically dominant. However, pre-administration of GHRP-6 brought the ratio to a level similar to that of WT mice. Although ghrelin is known to have various physiological effects (32–34), it is possible that during fasting, ghrelin may induce the whole body to a resting state by inhibiting the sympathetic nerve activity, and may act to maintain homeostasis based on the induction and maintenance of torpor. Indeed, as we have shown, ghrelin suppresses sympathetic nerve activity input to brown adipose tissue during torpor induction (1).

At this time, a decrease in serum noradrenaline concentrations was observed in C57BL/6J mice treated intraperitoneally with ghrelin, along with activation of neurons localized in the solitary nucleus (1). We were also able to show that serum noradrenaline levels were higher in *ghrl^-/-^* mice than in WT mice during torpor (1). In addition, another group of researchers reported that stimulation of the solitary nucleus by ghrelin reduced mean blood pressure and heart rate via inhibition of sympathetic nerve activity (29). These facts may suggest that the effect of ghrelin on blood pressure during torpor is a suppression of sympathetic nerve activity mediated by the solitary nucleus.

In this study, we also found that ghrelin not only inhibits sympathetic activity but also promotes parasympathetic activity. It has been suggested that intravenous ghrelin administration in humans may inhibit cardiac sympathetic activity and stimulate cardiac parasympathetic activity (35). Our results support their conclusion and indicate that endogenous ghrelin, which is elevated during torpor induction, may have similar effects. Future neuroendocrine analysis will be needed to elucidate the mechanism by which ghrelin lowers blood pressure during torpor.

Recently, Mohr et al. (36) show that the thirteen-line squirrels (*Ictidomys tridecemlineatus*), which endure the entire hibernation season without food, have only negligible hunger drive during an active-like interbout arousal state, even though plasma ghrelin levels are at levels that can induce feeding. These squirrels exhibit reversible inhibition of the hypothalamic feeding center, such that hypothalamic arcuate nucleus neurons exhibit reduced sensitivity to the orexigenic and anorexigenic effects of ghrelin and leptin, respectively (36). The results of this report imply that feeding is suppressed in these squirrels to maintain hibernation. Although the mechanism by which ghrelin secreted during fasting switches between feeding and resting periods needs to be elucidated in the future, the fact that ghrelin induces torpor during fasting may be one of the major roles of ghrelin as an anabolic hormone.

In summary, ghrelin secreted during fasting suppresses the sympathetic activity and actively induces a decrease in blood pressure. This change occurs simultaneously with the ghrelin-induced decrease in body temperature during fasting, which we previously reported (1), indicating that ghrelin is an essential hormone in the induction and maintenance of torpor, which is characterized by an active decrease in body temperature and blood pressure. Further elucidation of the mechanism of the torpor phenomenon may allow for future artificial control of torpor.

## Data Availability

The datasets presented in this paper can be found in online repositories. The names of the repository/repositories and their accession numbers (s) can be found in the article/**Supplementary Material**. Requests to access the datasets should be directed to satou_takahiro@kurume-u.ac.jp.

## Supplementary material

Supplementary Figure 1 Diurnal rhythm of heart rate in *ghrl^-/-^* mice. **(A)** Two-day heart rate changes for representative individual mice. The black bar shown on the horizontal axis represents the dark phase, and similarly the white bar represents the light phase. The orange and light blue backgrounds shown in the graph indicate the range over which data were used to draw the histogram in **(B)**. **(B)** Histograms of heart rate during the light phase (8:00 - 18:00; data obtained from orange background in **(A)**) and dark phase (20:00 - 6:00 the next day; data obtained from light blue background in **(A)**) of *ghrl^-/-^* mice. Data are means ± SEM (n = 5). ^*^P<0.05 (higher frequency in WT mice compared to *ghrl^-/-^* mice), ^†^P<0.05 (higher frequency in *ghrl^-/-^* mice compared to WT mice).

Supplementary Figure 2 Heart rate changes in *ghrl^-/-^* mice following fasting. *Ghrl^-/-^* +GHRP-6 indicates that the *ghrl^-/-^* mice were administered the ghrelin receptor agonist, GHRP-6, with an osmotic mini-pump, and Re-fed indicates re-feeding. The black bar shown on the horizontal axis represents the dark phase, and similarly the white bar represents the light phase. **(A)** Heart rate changes after fasting in representative individual mice. **(B)** Range of change in hourly means heart rate data from the start of fasting. Data are means ± SEM (n = 5). ^*^P<0.05 (comparison between WT mice, *ghrl^-/-^* mice, and *ghrl^-/-^* mice treated with the ghrelin receptor agonist GHRP-6).
