# Supplementary figures and images for: Ghrelin is essential for lowering blood pressure during torpor
Source: Front Endocrinol (Lausanne). 2024 Oct 10;15:1487028. doi: 10.3389/fendo.2024.1487028 (PMC11499174; doi:10.3389/fendo.2024.1487028)

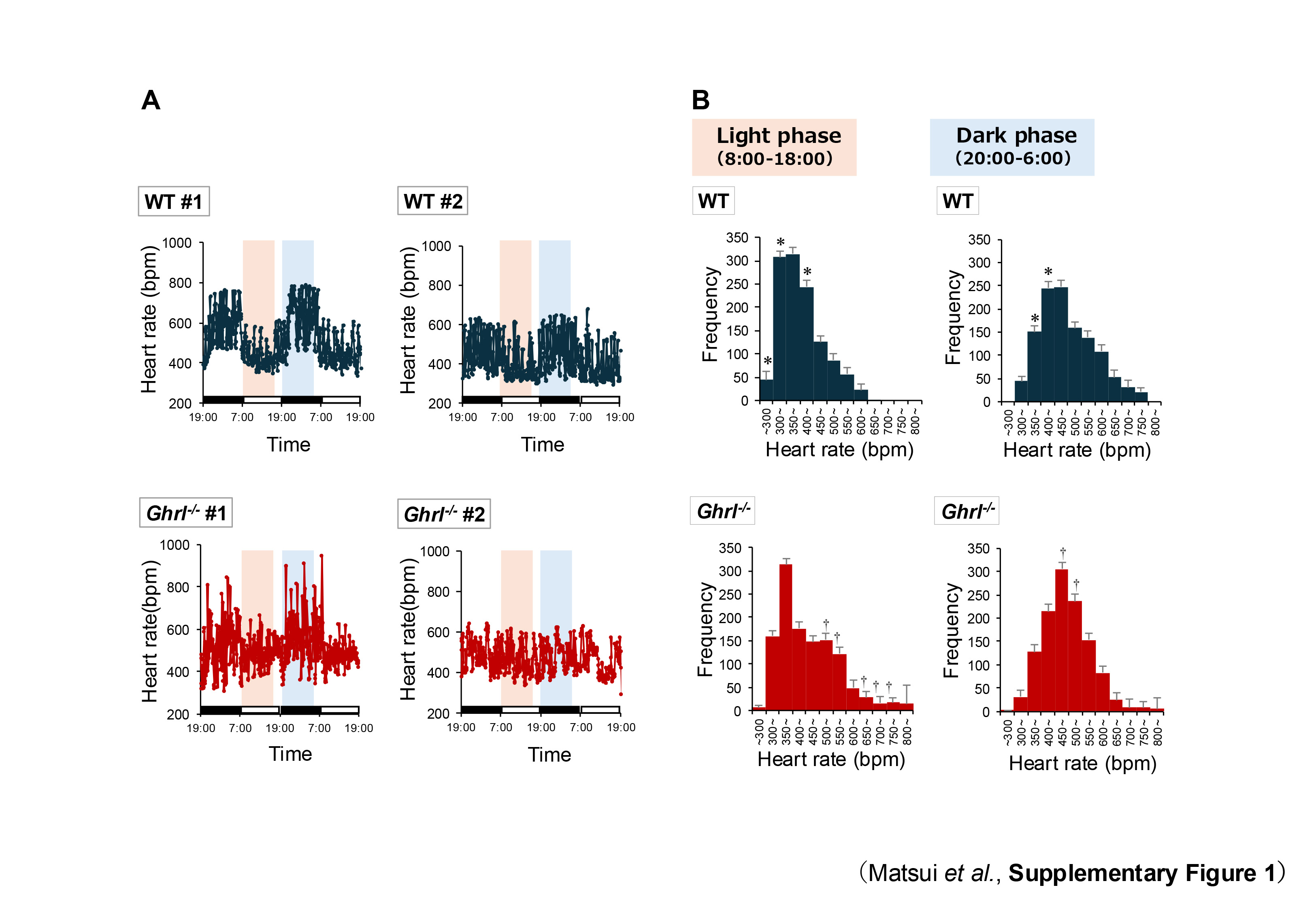

Supplement: Supplementary Figure 1 — Diurnal rhythm of heart rate in ghrl-/- mice. (A) Two-day heart rate changes for representative individual mice. The black bar shown on the horizontal axis represents the dark phase, and similarly the white bar represents the light phase. The orange and light blue backgrounds shown in the graph indicate the range over which data were used to draw the histogram in (B). (B) Histograms of heart rate during the light phase (8:00 - 18:00; data obtained from orange background in (A)) and dark phase (20:00 - 6:00 the next day; data obtained from light blue background in (A)) of ghrl-/- mice. Data are means ± SEM (n = 5). *P<0.05 (higher frequency in WT mice compared to ghrl-/- mice), †P<0.05 (higher frequency in ghrl-/- mice compared to WT mice). [file Image1.jpeg]

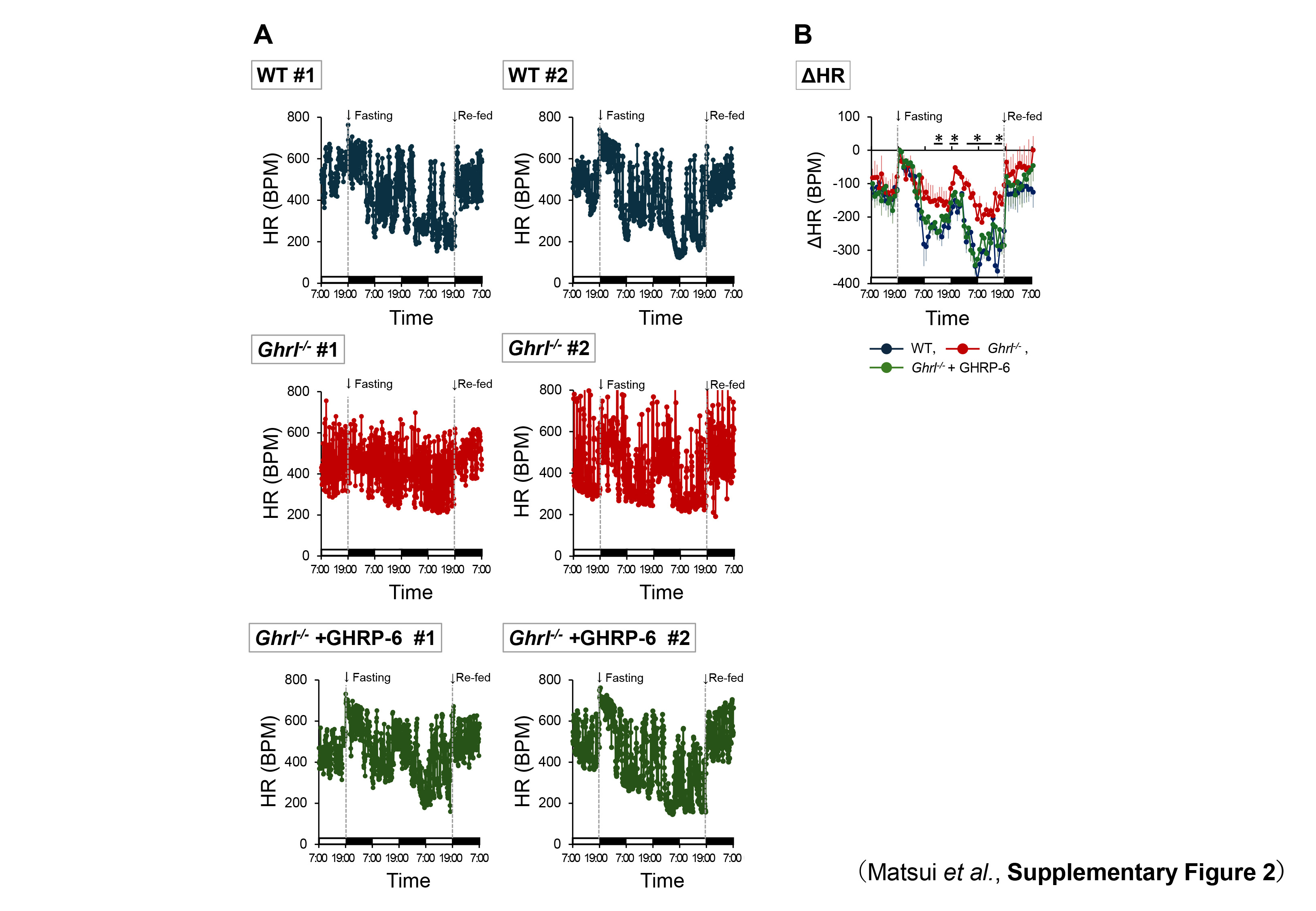

Supplement: Supplementary Figure 2 — Heart rate changes in ghrl-/- mice following fasting. Ghrl-/- +GHRP-6 indicates that the ghrl-/- mice were administered the ghrelin receptor agonist, GHRP-6, with an osmotic mini-pump, and Re-fed indicates re-feeding. The black bar shown on the horizontal axis represents the dark phase, and similarly the white bar represents the light phase. (A) Heart rate changes after fasting in representative individual mice. (B) Range of change in hourly means heart rate data from the start of fasting. Data are means ± SEM (n = 5). *P<0.05 (comparison between WT mice, ghrl-/- mice, and ghrl-/- mice treated with the ghrelin receptor agonist GHRP-6). [file Image2.jpg]
